# Development and antitumor activity of a BCL-2 targeted single-stranded DNA oligonucleotide

**DOI:** 10.1007/s00280-014-2476-y

**Published:** 2014-05-16

**Authors:** Wendi V. Rodrigueza, Michael J. Woolliscroft, Abdul-Shukkur Ebrahim, Robert Forgey, Patrick J. McGovren, Gerold Endert, Andreas Wagner, Danielle Holewa, Amro Aboukameel, Richard D. Gill, Charles L. Bisgaier, Richard A. Messmann, Christopher E. Whitehead, Elzbieta Izbicka, Robert Streeper, Michael C. Wick, Gabriela Stiegler, C. A. Stein, David Monsma, Craig Webb, Mina P. Sooch, Steffen Panzner, Ramzi Mohammad, Neal C. Goodwin, Ayad Al-Katib

**Affiliations:** 1 ProNAi Therapeutics, Inc., Plymouth, MI USA; 2 Wayne State University, Detroit, MI USA; 3 Novosom AG, Halle, Germany; 4 Polymun Scientific GmbH, Klosterneuburg, Austria; 5 BTNS, LLC, San Antonio, TX USA; 6 South Texas Accelerated Research Therapeutics (START), LLC, San Antonio, TX USA; 7 Montefiore Medical Center and the Albert Einstein College of Medicine, New York, NY USA; 8 Van Andel Research Institute, Grand Rapids, MI USA

**Keywords:** PNT2258, DNAi^®^, BCL-2, Liposomes, Anticancer agent, Deoxyribonucleic acid interference

## Abstract

PNT100 is a 24-base, chemically unmodified DNA oligonucleotide sequence that is complementary to a region upstream of the BCL-2 gene. Exposure of tumor cells to PNT100 results in suppression of proliferation and cell death by a process called DNA interference. PNT2258 is PNT100 that is encapsulated in protective amphoteric liposomes developed to efficiently encapsulate the PNT100 oligonucleotide, provide enhanced serum stability, optimized pharmacokinetic properties and antitumor activity of the nanoparticle both in vivo and in vitro. PNT2258 demonstrates broad antitumor activity against BCL-2-driven WSU-DLCL2 lymphoma, highly resistant A375 melanoma, PC-3 prostate, and Daudi-Burkitt’s lymphoma xenografts. The sequence specificity of PNT100 was demonstrated against three control sequences (scrambled, mismatched, and reverse complement) all encapsulated in a lipid formulation with identical particle characteristics, and control sequences did not demonstrate antiproliferative activity in vivo or in vitro. PNT2258 is currently undergoing clinical testing to evaluate safety and antitumor activity in patients with recurrent or refractory non-Hodgkin’s lymphoma and additional studies are planned.

**Electronic supplementary material:**

The online version of this article (doi:10.1007/s00280-014-2476-y) contains supplementary material, which is available to authorized users.

## Introduction

BCL-2, a member of the antiapoptotic protein family, plays a key and central role in preventing cell death, a defining characteristic of malignant cells [1]. BCL-2 confers an antideath phenotype and its overexpression contributes to the genesis of hematopoietic and lymphatic cancers [2–6]. Aberrant BCL-2 expression is driven by *t*(14;18) chromosomal rearrangement of the BCL-2 gene in many follicular (FL) and diffuse large B cell (DLBCL) lymphomas [3–6]. In chronic lymphocytic leukemia, impaired degradation of BCL-2 mRNA is linked to nucleolin and/or microRNA expression, resulting in continuous production of BCL-2 protein and the subsequent survival of leukemic cells [7, 8]. In cancers of the breast, skin, prostate, sarcomas, and lung, BCL-2 is implicated in the development of chemo-resistance [9]. Given its biological importance, BCL-2 is a desirable target for therapeutic development. Numerous approaches have been reported to block or modulate the production of BCL-2 at the DNA level (e.g., retinoids and histone deacetylase inhibitors), at the RNA level (targeted antisense oligonucleotides, siRNA or miRNA), or the protein level (e.g., pan inhibitors of BH3 family members of BCL-2, or recently the specific BCL-2 inhibitor, ABT-199) reviewed in [10].

There is an emerging understanding that CpG islands surround mammalian cell promoter regions and that these non-methylated regions contribute to gene regulation [11, 12]. The recognition that these genomic regions are DNAse I-hypersensitive enabled the discovery of cis-regulatory elements that act as transcription factors, enhancers, silencers, repressors, or control regions, which regulate gene expression [13–15]. Additionally, higher-order secondary structures (quadruplexes, cruciforms, or I-motifs) that surround the promoter regions of oncogenes may also serve as cis-regulatory domains to modulate transcription [16, 17]. It has been demonstrated that exposing cells to short DNA sequences containing these motifs reduced mRNA and protein levels [18, 19]. Others have recognized the importance of regulatory regions specific for BCL-2 [20–23]. Young and Korsmeyer demonstrated that a series of 20 base deletions between the P1 and P2 promoter of BCL-2 decreased transcription. Miyashita et al. reported that p53-dependent regions upstream of the BCL-2 gene act as negative regulatory elements, and Duan et al. showed long-range regulatory effects on BCL-2 transcription by enhancers in the IgH 3’ region. The observations support the hypotheses that these regulatory regions may be favorable therapeutic targets for hybridization due to the accessible chromatin state during oncogene up-regulation and transcription.

We describe a novel approach to blocking transcription termed DNA interference (DNAi^®^). DNAi therapeutic candidates are a new class of nucleic acid-based drugs. They are single-stranded sequences of unmodified phosphodiester DNA having lengths of 20–34 bases. These sequences are designed to be complementary to non-coding, non-transcribed regions of genomic DNA upstream of gene transcription start sites. The hybridization of the DNA-interfering oligonucleotide to its targeted region results in gene modulation with phenotypic changes and a modulation of mRNA and protein levels. While DNAi against BCL-2 is described in this paper, DNAi oligonucleotides may be designed to also target other regions of the genome to modulate genes. We describe here the development of PNT2258, containing PNT100, a 24-base single-stranded DNA oligonucleotide specific to BCL-2 encapsulated in amphoteric liposomes. The liposomes protect the oligonucleotides from nuclease degradation, facilitate cellular uptake, and enable endosomal escape to the nucleus, where the biological effects of the oligonucleotides occur [24–26]. PNT2258 is currently undergoing clinical development against BCL-2-driven malignancies [27].

## Materials and methods

### PNT100 and control DNAi oligonucleotides

Oligonucleotides were produced by multi-step solid-phase organic synthesis involving on-column cleavage from solid support, base de-protection, followed by ion exchange (IEX) purification, ultrafiltration/diafiltration, concentration, and freeze-drying. The PNT100 and controls include: PNT100, 5′-CACGCACGCGCATCCCCGCCCGTG-3′; methylated PNT100, 5′-CAXGCAXGXGCATCCCXGCCXGTG-3′, where X represents a methylated cytosine base; scrambled control, 5′-CGGCGTGCACCCCACCCACGCCGT-3′; reverse complement control, 5′-CACGGGCGGGGATGCGCGTGCGTG-3′, which is the reverse sequence of PNT100 and is 100 % homologous with the coding strand; mismatched control, 5′-CACGCACGCGCATCCTTGCCCGTG-3′ or 5′-CACGCACGCGCATCCTTGCCCATG-3′; randomer, 5′-NNNNNNNNNNNNNNNNNNNNNNNN-3′, where N represents a wobble; PNT100cy 5′-CACGCGCGCGCATCCCCGCCCGTG-3′. Oligonucleotides were purchased from TriLink, Dow, Sigma, or NITTO DENKO Avecia.

### Lipids and transfection agents

NeoPhectin AT containing the cationic cardiolipin 1, 3-Bis-(1,2-bis-tetradecyloxy-propyl-3-dimethylethoxyammoniumbromide)-propane 2-ol was purchased from NeoPharm (Waukegan, IL). 1-Palmitoyl-2-oleoyl-sn-glycero-3 phosphocholine (POPC) and 1,2-dioleoyl-sn-glycero-3-phosphoethanolamine (DOPE) were purchased from Lipoid GmbH or Avanti Polar Lipids. Cholesteryl hemisuccinate (CHEMS) and cholesteryl-4-[[2-(4-morpholinyl)ethyl]amino]-4-oxobutanoate (MOCHOL) were produced by Merck & Cie (Shaffhuasen, Switzerland).

### Preparation of encapsulated oligonucleotides and PNT2258

During preliminary screening, oligonucleotides were encapsulated in a variety of liposome compositions, including the cationic NeoPhectin AT system and into various amphoteric liposomes denoted as SMARTICLES^®^ (from Novosom AG, now Marina Biotech). While NeoPhectin spontaneously forms liposomes upon mixing with oligonucleotides, the other lipids that generate the amphoteric liposomes were mixed in ethanol and combined with an acidified (pH 4) aqueous solution of each oligonucleotide tested. The mixing process with the latter results in encapsulation in a 30 % ethanol suspension followed by dilution in an excess of phosphate-buffered saline with simultaneous neutralization to pH 7.5 [26, 28, 29]. For PNT2258, a cross-flow injection technique was utilized to enable continuous mixing of the lipid ethanolic solution with aqueous PNT100 to encapsulate PNT100 in liposomes followed by an immediate pH shift to neutralize the mixture. Ethanol was removed by dialysis or diafiltration to exchange saline with sucrose (phosphate-buffered sucrose), followed by sterile filtration and filling into glass vials.

### Characterization of the physicochemical properties of formulation solutions and dosing solutions of oligonucleotides

OD260-derived concentrations, representing total oligonucleotide content, were used to compare encapsulated oligonucleotide dosing solutions used for animal studies with PNT100, control oligonucleotides, and prototype PNT2258 formulations. Standard curves with each oligonucleotide were used along with spectral scans to confirm the OD260 contribution resulted from the oligonucleotides and not the liposome/lipid nanoparticles. The percent unencapsulated oligonucleotide was determined by OD260 following ultrafiltration using Centrisart 100 K cutoff membranes. Where indicated, the PNT2258 concentrations were reported as PNT100 content after correcting for purity as determined by ion exchange (IEX) chromatography. Reverse-phase HPLC was used to quantify lipids in the nanoparticles. Particle diameters and zeta potentials were determined by dynamic light scattering using a Malvern Nano ZS (Malvern, PA).

### Cell culture and treatment with DNAi

Cell lines were maintained in suspension or monolayer cultures in media supplemented with 10 % fetal bovine serum (FBS). Breast (MDA-MB-231, BT-474, and T47D), melanoma (A375 and M14), prostate PC-3, lymphoma (Daudi-Burkitt’s, SU-DHL-6, and Pfeiffer), and mouse mammary NMuMG lines were purchased from ATCC^®^. WSU-DLCL2 and WSU-FSCCL lines were purchased from DSMZ GmbH (Germany) or provided by Ramzi Mohammad of Wayne State University. The cell lines were authenticated by the providers and were maintained under the recommended conditions for propagation and experimental use.

Unless otherwise noted, cell exposure studies utilized methylated oligonucleotides without formulation. For adherent lines, cells were seeded in 6- or 24- well plates or T-25 flasks (Corning Life Sciences) at 2.0 × 10^5^ cells per flask in 5 mL media. One day after passage, the medium was replaced with fresh media containing the test oligonucleotides. Cultures were incubated at 37 °C in a humidified atmosphere of 5 % CO_2_. Cells were washed with 1× PBS and incubated with 0.25–1 % trypsin and 0.02 % EDTA to disperse the cells. The number of living and dead cells was assessed following 0.1 % trypan blue exposure, with the percentage of inhibition reported as a percent of live cells present in saline-treated controls. The effects of treatment on proliferation were assessed using MTT assay with 2,500 cells per well seeded in 96-well plates. Suspended cells were grown in 24-well plates and treated as described above. For PC-3 exposure studies, oligonucleotides were formulated with NeoPhectin. Typically, cells were exposed for 30 min or 6 h, washed, then the effects of treatment on proliferation were assess by CellTiter-Glo^®^ (Promega) 48 h post-treatment or BCL-2 and GAPDH expression was measured by qPCR.

### Pharmacokinetics, pharmacology, tissue distribution, and xenograft studies

#### Pharmacokinetic studies and xenograft animal studies

PNT2258 and its prototypes were evaluated as single agents or in combination in four different human tumor xenograft model systems. Two of the models were non-Hodgkin’s lymphoma models (WSU-DLCL2 (a diffuse large B-cell) and Daudi-Burkitt’s). The other xenograft models included A375 melanoma and the PC-3 hormone refractory prostate carcinoma. For WSU-DLCL2, female ICR SCID mice (Taconic) or female C.B-17 SCID mice were implanted subcutaneously with donor WSU-DLCL2 xenograft fragments in their flanks. A parallel set of WSU-DLCL2 xenograft mice were also used for tissue distribution studies. For Daudi-Burkitt’s, female C.B-17 SCID mice were implanted subcutaneously with 1 × 10^7^ Daudi cells in their flanks. A parallel set of Daudi-Burkitt’s xenograft mice were also used for pharmacokinetic and tissue distribution studies. For A375, female nu/nu mice were implanted subcutaneously in the flank with 1 mm^3^ fragments. For PC-3, male SCID/NCr mice were subcutaneously implanted with 5 × 10^6^ PC-3 cells. All xenograft tumor models were conducted through contract or research collaborations at MPI Research (PC-3), South Texas Accelerated Research Therapeutics (A375 and WSU-DLCL2), Piedmont Research (Daudi and A375), Karmanos Cancer Center (WSU-DLCL2), and Van Andel Research Institute (PC-3). BALB/C mice were purchased from Charles River Labs and used for pharmacokinetic studies. Test samples were provided in a blinded manner. All protocols and procedures employed in this work were approved by each testing centers’ Institutional Animal Care and Use Committee (IACUC).

#### Animal monitoring, tumor measurement, and data calculation

Upon attaining tumor volumes of 100–200 mm^3^, animals were randomized into treatment groups. Thereafter, clinical signs, tumor measurements, and body weights were recorded 3–5 times per week. Tumor volume was calculated using the formula (*l* × *w* ^2^)/2, where *l* and *w* are the length and width of the tumor, respectively. Animals were euthanized when tumor sizes reached 1,000 mm^3^, 2,000 mm^3^ or at approximately 60 days depending on the research organizations’ approved protocols. Efficacy endpoints, including time to tumor endpoint, tumor growth delay, and net log_10_ cell kill, were calculated as follows using previously described methods [30]. Gross cell kill was calculated using the following formula: [T-C (days)]/(3.32 * Td), where T-C is the tumor growth delay and Td is the tumor volume doubling time (days). Individual tumor volumes which decreased to <50 % of their volumes at treatment initiation for three consecutive measurements were considered partial regressions (PR). Individual tumor volumes that were not measurable for three consecutive measurements were considered complete regressions (CR). Complete regressions persisting until the end of the study were considered tumor-free survivors (TFS). Data and statistics were analyzed using Prism 5.0 (GraphPad; San Diego, CA) and Microsoft Excel.

#### Plasma measurement of PNT2258 by hybridization–ligation and plasma immune markers

Whole blood was collected in K_2_EDTA-coated tubes, placed on ice, centrifuged to obtain plasma and stored at −80 °C until analyses. Samples were treated with 10 % (v/v) Tween-20 detergent and heated to 90 °C to liberate PNT100 from PNT2258 then diluted fourfold with a template probe (complementary and specific to the entire sequence of PNT100) labeled with biotin on its 3′-end and a 9-mer overhang to the opposing end. The solution was incubated at 37 °C for 1 h in NeutrAvidin-coated plates, prior to the addition of a mixture containing a digoxigenin-label signal probe which ligates the 3′ terminus of PNT100 with the 5′end of the ligation probe. Unbound ligation probe was washed away prior to antibody (targeting digoxigenin) addition and conjugation to alkaline phosphatase. AttoPhos^®^ substrate was added and the reaction terminated with EDTA solution prior to fluorescent signal measurement (excitation: 435 nm; emission: 555 nm). The lower limit of quantitation (LLOQ) was 3 ng/mL using PNT2258 as a standard. Multiplex immunoassays of mouse plasma obtained from WSU-DLCL2-tumored animals 8 h post-PNT2258 dose were assayed in triplicate per the Affymetrix Procarta Mouse 37-plex kit protocols (Fremont, CA) and visualized using a Luminex 100 IS System (Luminex Corporation, Austin, TX). Analyte concentrations were calculated from the standard curves using Bio-Plex Manager 4.1.1 (Bio-Rad Laboratories, Hercules, CA). Statistical analysis was done using Student t statistic; *P* values <0.05 were considered significant.

#### Pharmacodynamic sampling of tumors and PNT2258 tissue levels

Tumors and organs were collected, snap frozen, weighed, and stored until analyses. Tissue levels of PNT2258 were assessed by two independent labs, Charles River Labs and Helix Diagnostics, using the hybridization–ligation assay described above for plasma analyses (LLOQ: 50 μg/g of tissue) or by capillary gel electrophoretic detection (LLOQ: 5 ng/g of tissue). PNT2258 levels in xenograft tumors were measured through direct hybridization with capture and extender probes that recognize only PNT100 amidst the total RNA extract (LLOQ 300,000 copies of PNT100). Tumor homogenates were prepared by pulverizing tumors under liquid nitrogen, followed by homogenization in 900 μL of homogenizing solution (Affymetrix) supplemented with 9 μL of proteinase K (50 mg/mL). The homogenates were incubated at 65 °C for 30 min, then clarified by centrifugation, and stored at −70 °C until analyses. Tumors excised from animals treated with PNT100 formulated with NeoPhectin, PNT100R formulated with NeoPhectin, NeoPhectin alone, or sucrose (vehicle control) were pooled into groups, dissected mechanically into single cell suspensions, and subjected to protein analysis by Western blot using antibodies (Santa Cruz Biotechnology) along with total protein quantitation by BCA assay (Pierce).

## Results

### Demonstration of antiproliferative and cytotoxic effects of DNAi oligonucleotides and selection of PNT100

Oligonucleotide sequences targeted along the 5′ region upstream of the BCL-2 ATG start site were synthesized and tested for antiproliferative activity against breast and melanoma cell lines. The oligonucleotides ranged from 20 to 26 bases in length, contained 61–100 % CG content, and mapped within the region translocated during the t(14;18) rearrangement. The oligonucleotides were methylated at the cytosine bases when used in cell exposure studies. The sequences were identical to the non-template strand and 100 % complementary to the template strand of genomic DNA. Of the oligonucleotides tested, a 24-base sequence of 5′-CAXGCAXGXGCATCCCXGCCXGTG-3′ showed the broadest antiproliferative effects against a panel of breast (67–76 % inhibition) and melanoma (42–65 %) cell lines (see Supplemental Figure S1A). This oligonucleotide also showed robust and time-dependent cytotoxic effects in WSU-FSCCL (see Supplemental Figure S1B), a fast growing BCL-2 and CMYC-driven non-Hodgkin’s follicular lymphoma cell line characterized by the BCL-2 t(14;18)(q32;q21) and CMYC t(8;11)(q24;q21) rearrangements [31]. Furthermore, BL2 (which represents methylated PNT100) and BL7 were counterscreened against an immortalized normal mouse mammary gland cell line (NMuMG, see Supplemental Figure S1C). BL2 (mePNT100) did not result in cytotoxicity at either 24 or 96 h post-exposure, whereas BL7 showed significant cytotoxicity at 96 h post-exposure. The lack of cytotoxicity in BL2 suggests specificity for the human sequence. This 24-base sequence was nominated as a lead BCL-2 therapeutic candidate and further tested for sequence specificity.

### In vitro sequence specificity testing of PNT100

**Fig. 1:**
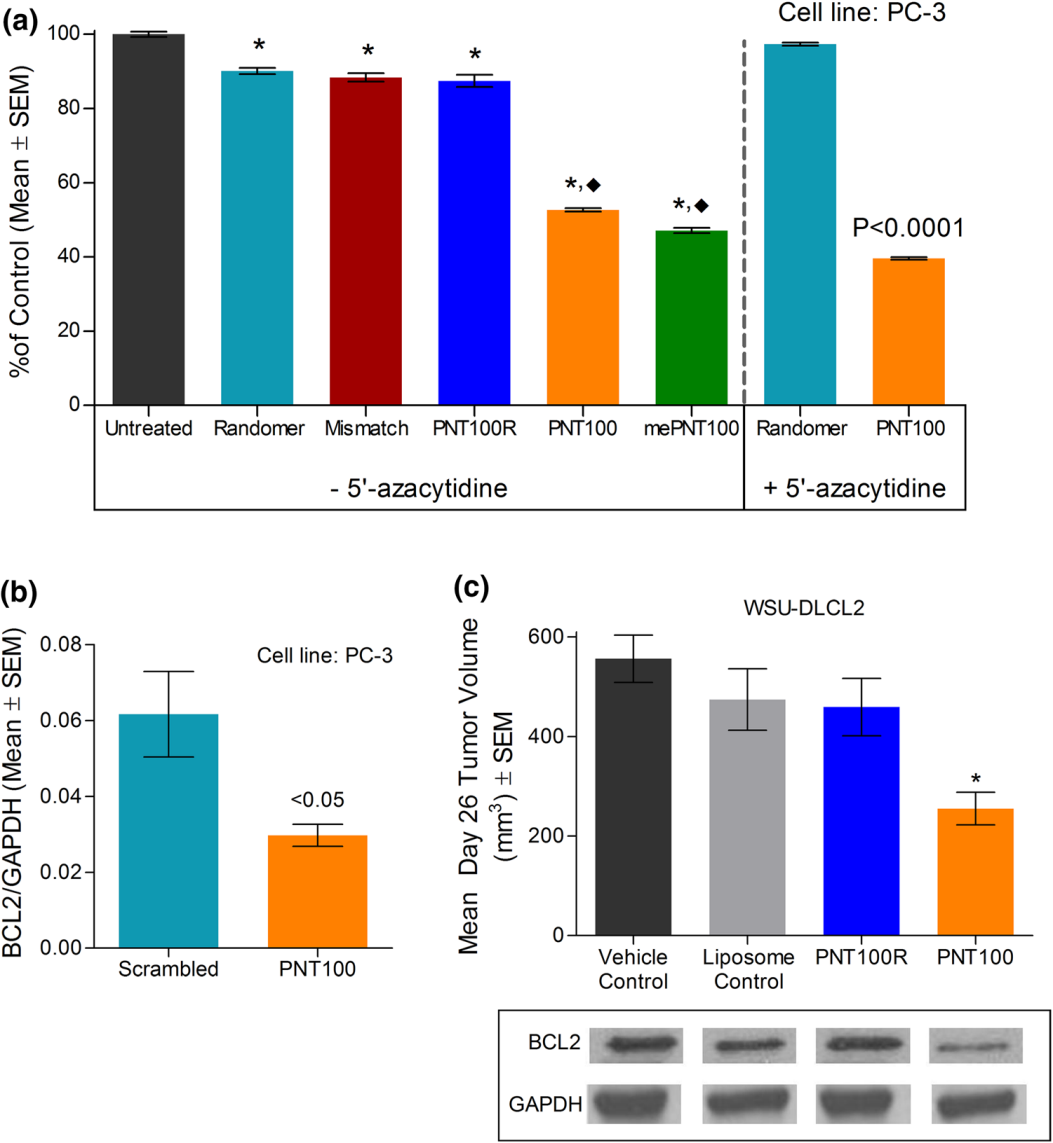
Effect of PNT100 or controls formulated with NeoPhectin on cell proliferation, BCL-2 expression, and antitumor activity. **a** *Left panel* PC-3 prostate cancer cells were exposed to 10 μM PNT100 (triplicate per group), methylated PNT100, and controls. After 6 h of exposure, cells were washed and allowed to equilibrate and analyzed 48 h post-exposure by Cell Titer-Glo assay. *P < 0.001 versus control, ^♦^ *P* < 0.001 versus scrambled, mismatched, randomer, and reverse control (PNT100R). **a** *Right panel* in a separate experiment, PC-3 prostate cancer cells were exposed to 5 μM 5′-azacytidine in combination with 10 μM PNT100, randomer or scrambled control. After 72 h of exposure, cells were analyzed for viability by Cell Titer-Glo assay. *P* < 0.0001 versus scrambled or randomer control. **b** BCL-2 levels in PC-3 prostate cancer cells exposed to PNT100 and the scrambled control (10 μM). After 6 h of exposure, BCL-2 and GAPDH RNA expressions were analyzed via qPCR. *P < 0.05 versus scrambled control. **c** Mean tumor size in animals following intravenous administration (IV, via tail vein) of 10 mg/kg PNT100 (NeoPhectin PNT100), reverse control (NeoPhectin PNT100R) or the vehicle controls (saline or NeoPhectin liposomes). WSU-DLCL2 tumor xenografts were implanted subcutaneously into animals and allowed to grow to an average of 100 mm^3^ size prior to treatment with five daily doses. Animals (*n* = 8 for all groups) were killed 26 days post-dosing, mean tumor weights measured, and BCL-2 protein levels were analyzed using protein immunoblots. *P < 0.05 versus Saline control, NeoPhectin Liposome control, and NeoPhectin PNT100R

To determine whether methylation was necessary for antiproliferative and cytotoxic effects, mePNT100 and unmethylated PNT100 were combined with a transfection agent, NeoPhectin AT, and the cytotoxic effects against PC-3 cells were tested (see Fig. 1a). The results showed equal activity between mePNT100 and PNT100, with approximately a 50 % reduction of cell proliferation post-exposure. Furthermore, the activity of PNT100 was compared to randomer, mismatched, and a reverse control of PNT100 (PNT100R) oligonucleotide controls. With the exception of the scrambled control, all sequences tested had identical 3’termini to control for any non-specific toxicity that has been reported due to the release of terminal bases [32]. Data show minimal antiproliferative effect of these oligonucleotides when compared to saline control (see Fig. 1a), with PNT100 or mePNT100 having the greatest effect. The activity was not influenced by the presence of 5-azacytidine, an inhibitor of DNA methyltransferase activity (Fig. 1a, right panel), indicating that the effect was not methylation dependent [33]. Further, BCL-2 expression following exposure with PNT100 was compared to the scrambled control (Fig. 1b).

To develop PNT100 as an intravenous drug candidate, liposomes were chosen to protect the unmodified oligonucleotide, enhance its pharmacokinetic profile, and facilitate cellular delivery. Initially, NeoPhectin was evaluated. The lipid components in NeoPhectin are similar to LErafAON, a liposome-encapsulated antiraf antisense oligonucleotide developed by NeoPharm [34]. PNT100 formulated with NeoPhectin demonstrated antitumor activity against WSU-DLCL2 xenografts and reduced BCL-2 expression in excised tumors when assessed 26 days post-treatment (Fig. 1c) when compared to liposome controls (NeoPhectin alone) or the reverse sequence of PNT100 formulated with NeoPhectin (NeoPhectin PNT100R). PNT100 formulated with NeoPhectin (NeoPhectin PNT100), however, did not demonstrate consistent stability when exposed to serum or physicochemical characteristics suitable for development as an oncology therapeutic. Therefore, candidates for a new delivery system were screened.

### Evaluation of amphoteric liposomes and optimization

**Fig. 2:**
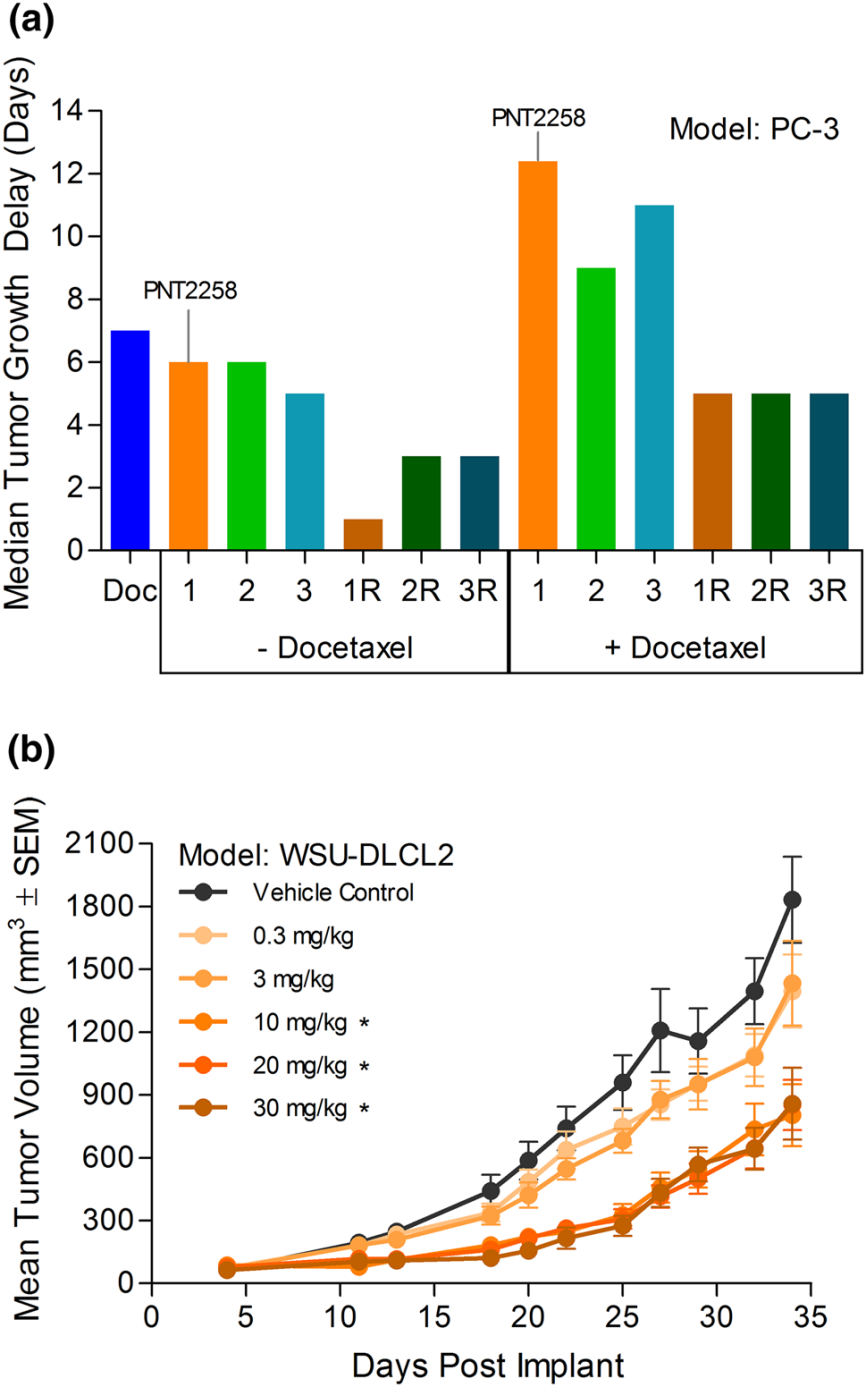
Antitumor activity against xenograft tumors of several formulations of PNT100 or PNT100R encapsulated in SMARTICLES (amphoteric liposomes) and the dose response of PNT100 in the chosen formulation. **a** SCID/NCr mice were implanted with PC-3 cells and treated with vehicle (saline) control, PNT100R or PNT100 encapsulated in three different lipid compositions as a single agent or in combination with docetaxel. PNT100, PNT100R, and the vehicle control were administered IV at 10 mg/kg daily for 5 days. Docetaxel was dosed at 2 and 5 mg/kg on day 2 and 5, respectively. Bars represent the median tumor growth delay (*n* = 5 for all groups). Various liposome formulations with PNT100 are represented as numbers (i.e., 1, 2, and 3), while the corresponding formulations with PNT100R are designed with “R” (i.e., 1R, 2R, and 3R). Formulation 1 is PNT2258. **b** Dose response of prototype PNT2258 (PNT100 encapsulated in lipid composition chosen for PNT2258, pre-optimization). SCID mice were implanted with WSU-DLCL2 cells and administered IV with saline (vehicle control) or prototype PNT2258 at doses ranging from 0.3 to 30 mg/kg (based on OD260 total oligonucleotide content). Mean tumor volumes following treatment (*n* = 7 per group) are shown. *P < 0.05 versus vehicle control and prototype PNT2258 at 0.3 and 3 mg/kg. Improvements made from pre-optimization to post-optimization resulted in an increase in tumor growth delay from 9 to 21 days and an increase in net cell kill from 0.2 to 1.1 using the same dose (10 mg/kg) and schedule (QDx5)

The SMARTICLES^®^ platform represents a series of liposomes having amphoteric properties (i.e., pH-dependent prevalence of either acidic or basic groups) that may be modulated across physiological pH ranges. This platform showed promising serum stability and proven tissue delivery with broad cellular localization, including nuclear delivery [26]. A number of lipid compositions including pH-sensitive cationic or anionic, fusogenic, and bilayer/stabilizing lipids were tested. Formulations that met criteria of >50 % encapsulation, <15 % serum release, <200 nm particle size, and <0.2 poly-dispersity underwent further testing in xenograft models. Encapsulated PNT100 and PNT100R were tested for antitumor activity against PC-3 xenografts as single agents and in combination with docetaxel (Fig. 2a). As single agents, all formulations containing PNT100 showed activity (labeled as 1, 2, or 3) with formulation 1 and 2 showing greater activity than 3, but equivalent tumor growth delay. Less activity was observed with formulations containing PNT100R (labeled as 1R, 2R, or 3R). Formulation 1 was chosen because (1) it showed good single-agent activity and an additive effect with encapsulated PNT100 when combined with docetaxel and (2) the liposome formulation showed the least antitumor activity with PNT00R as a single agent or in combination with docetaxel. Therefore, based on the results from this activity screen and the physicochemical characterizations, a composition of POPC, DOPE, CHEMS, and MOCHOL at molar ratios of 6:24:23:47 was chosen for PNT2258.

Due to the robust and consistent single-agent activity observed, the drug product characteristics of PNT2258 were optimized in vivo against WSU-DLCL2 tumor xenografts. A broad dose range was tested (Fig. 2b). However, as seen, at doses of ~10 mg/kg, increasing the dose further did not produce additional antitumor activity, and increasing the dose even further produced frank toxicity in the experimental animals. These data suggested that the dose–response curve for PNT2258 is relatively flat in mice over the effective dose range and is consistent with dose-dependent pharmacokinetics and therapeutic efficacy often seen with liposome-delivered therapeutics once saturation or clearance blockade is achieved [36]. The effects of PNT100-to-lipid ratio, particle size, size distribution, and freezing on antitumor activity were testing iteratively in WSU-DLCL2-tumored mice and these are summarized in Table 1. Pre-optimization formulations administered IV for five consecutive days at a 10 mg/kg PNT100 equivalent dose (q5d) resulted in a TGD of 0–13 days and net log_10_ cell kills of 0–0.5 across the doses tested. Net kill is the change in tumor burden (logs) over the treatment period and enables the quantitative comparison of efficacy across experimental protocols by normalizing the efficacy data for treatment regimens of varied duration and differences in tumor growth rates between experiments or models. Positive values indicate a reduction of tumor burden occurred at the end of therapy relative to the pre-treatment burden. Post-optimization formulations (designated as PNT2255 or PNT2256) resulted in a TGD of 23–36 days, net log_10_ cell kills of 1–2, and a proportionate increase in complete responses and tumor-free survivors (Table 1). Of note, targeting average particle diameters of ~130 nm and changing PNT100-to-lipid ratios to 1:25 from 1:50 improved activity. Process refinements (mixing conditions, extrusion to refine particle size, and polydispersity) were applied, and these prototype formulations were tested at doses of 10 mg/kg to define the physicochemical characteristics of encapsulated PNT100. The efficacy of frozen *versus* refrigerated formulations of PNT2258 alone or in combination with rituximab showed that freezing the formulation demonstrated at least equivalent activity and equivalent physicochemical characteristics upon thawing when compared to the refrigerated formulation, indicating that freezing did not affect activity (Table 1). Therefore, freezing was implemented as the final step during manufacturing to enhance shelf life. The resulting nanoparticles with a diameter of approximately 130 nm encapsulating PNT100 at an oligonucleotide-to-lipid ratio of 1:25 (w/v) was designated as PNT2258.

**Table 1:** Formulation development and optimization of PNT2258

Tumor model	Formulation^a^	Dose (mg/kg)^b^	PNT2258 schedule^c^	Combo agent	Dose (mg/kg)	Combo agent schedule^c^	PR *N* (%)	CR *N* (%)	Tumor-free survivors *N* (%)	Tumor growth delay (days)	Net cell kill	Min % T/C
*Delivery vehicle*												
WSU-DLCL2	NeoPhectin	0.5	qd × 5	–	–	–	0 (0)	0 (0)	0 (0)	4	0.2	42
WSU-DLCL2	SMARTICLES	0.5	qd × 5	–	–	–	0 (0)	0 (0)	0 (0)	4	0.2	42
*PNT100*-*to*-*lipid ratio*												
WSU-DLCL2	PNT2253	0.3	qd × 5	–	–	–	0 (0)	0 (0)	0 (0)	0	−0.2	70
WSU-DLCL2	PNT2253	3	qd × 5	–	–	–	0 (0)	0 (0)	0 (0)	1	−0.2	59
WSU-DLCL2	PNT2253	10	qd × 5	–	–	–	0 (0)	0 (0)	0 (0)	7	0.1	36
WSU-DLCL2	PNT2253	20	qd × 5	–	–	–	0 (0)	0 (0)	0 (0)	9	0.2	23
WSU-DLCL2	PNT2254	0.3	qd × 5	–	–	–	0 (0)	0 (0)	0 (0)	2	−0.1	66
WSU-DLCL2	PNT2254	3	qd × 5	–	–	–	0 (0)	0 (0)	0 (0)	2	−0.1	71
WSU-DLCL2	PNT2254	10	qd × 5	–	–	–	0 (0)	0 (0)	0 (0)	8	0.1	32
WSU-DLCL2	PNT2254	20	qd × 5	–	–	–	0 (0)	0 (0)	0 (0)	9	0.2	32
WSU-DLCL2	PNT2254	30	qd × 5	–	–	–	0 (0)	0 (0)	0 (0)	9	0.2	23
*Particle diameter refinement and freezing*												
WSU-DLCL2	–	–	–	rituximab	20	Day 2 and 5	1 (13)	3 (38)	4 (50)	>58	3.6	0
WSU-DLCL2	Refrigerated (PNT2255)	10	qd × 5	–	–	–	0 (0)	1 (13)	0 (0)	13	0.5	20
WSU-DLCL2	Frozen (PNT2256)	10	qd × 5	–	–	–	1 (13)	2 (25)	2 (25)	21	1.1	2
WSU-DLCL2	PNT2255	10	qd × 5	rituximab	20	Day 2 and 5	0 (0)	1 (13)	5 (63)	>63	3.9	0
WSU-DLCL2	PNT2256	10	qd × 5	rituximab	20	Day 2 and 5	0 (0)	3 (38)	5 (63)	>63	3.9	0
*Dose schedule dependence*												
WSU-DLCL2	–	–	–	rituximab	2	Day 2 and 5	5 (71)	1 (14)	0 (0)	32	1.8	3
WSU-DLCL2	PNT2256	10	qd × 5	–	–	–	0 (0)	3 (43)	1 (14)	31	1.7	1
WSU-DLCL2	PNT2256	10	q3d × 3 weeks	–	–	–	0 (0)	0 (0)	0 (0)	9	−0.6	27
WSU-DLCL2	PNT2256	10	q2d × 2 weeks	–	–	–	0 (0)	0 (0)	0 (0)	7	−0.4	43
WSU-DLCL2	PNT2256	10	qdx5	rituximab	2	Day 2 and 5	0 (0)	2 (29)	2 (29)	38	2.1	0

^a^PNT225X formulations represent prototypes of PNT2258 encapsulated in MOCHOL/CHEMS/DOPE/POPC (47:23:24:7). PNT2253 = PNT100-to-lipid ratio of 1:50 and average particle diameter of ~90 nm; PNT2254 = PNT100-to-lipid ratio of 1:25 and average particle diameter ~90 nm; PNT2255 = Refrigerated prototype having PNT100-to-lipid of 1:25 and average particle diameter refinement to ~130 nm; PNT2256 = Frozen prototype having PNT100-to-lipid of 1:25 with average particle diameter refinement to ~130 nm

^b^Dose calculated based on total oligonucleotide content measured by OD260

^c^Schedule: qdX5 = five daily doses; q3d = a dose every three days; q2d = a dose every other day. *N* = number of animals; PR = partial responder; CR = complete responder

### Pharmacokinetics, tissue distribution, and immune evaluation of PNT2258

**Fig. 3:**
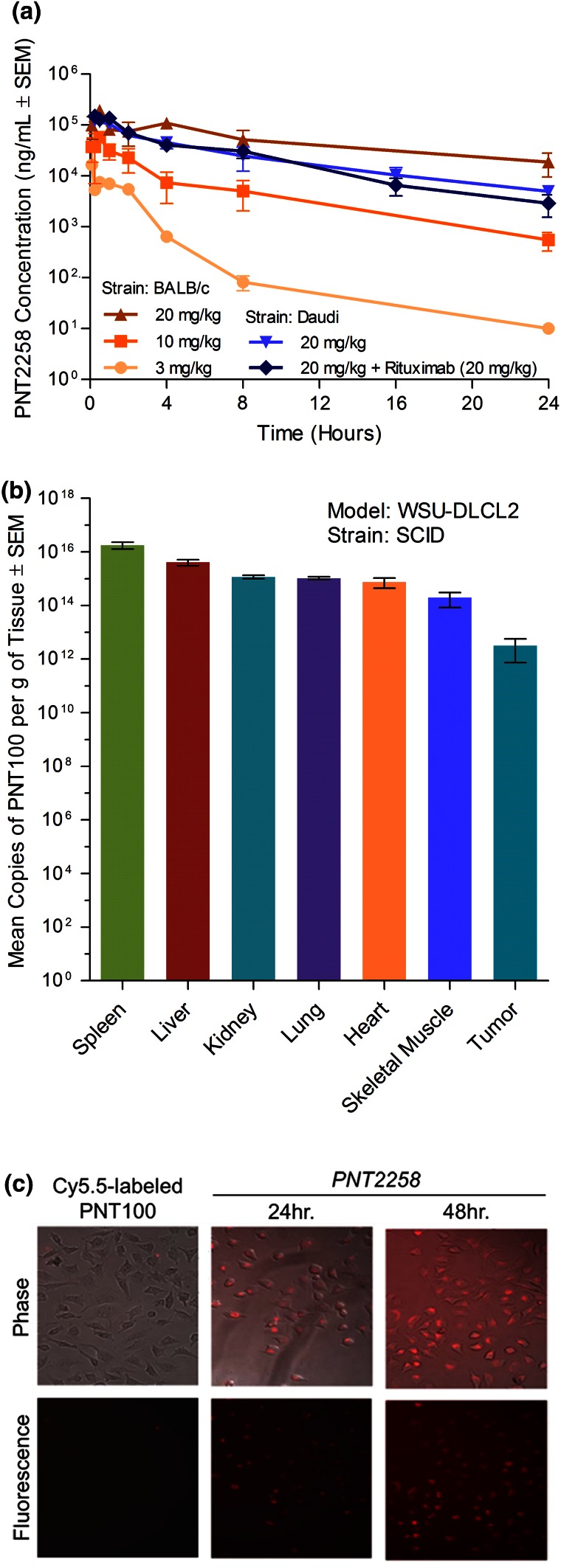
Pharmacokinetics, tissue distribution, and time course of cellular uptake of PNT2258. **a** Pharmacokinetics of PNT2258 in BALB/c or SCID Daudi-Burkitt’s-tumored mice measured in plasma by the hybridization–ligation assay. PNT2258 was administered IV as a single dose at 3, 10, or 20 mg/kg (*n* = 3/group) in BALB/c mice (data shown in *orange*). PNT2258 was administered as a single dose at 20 mg/kg as a single agent or in combination with a single dose of rituximab (20 mg/kg). *N* = 3 or 4 per group, data shown in *blue*. Both agents were administered IV within 5 min of each other. Blood was collected at 5, 15, 30 min, 1, 2, 4, 8, and 24 h and plasma was stored at −80 °C until analysis. **b** Tissue distribution of PNT2258 in WSU-DLCL2-tumored mice. Nude mice were implanted with WSU-DLCL2 cells and administered IV with 20 mg/kg PNT2258. Tissue samples were collected at 8 h after the initial treatment for analysis by gel capillary electrophoresis visualized by hybridization with a PNT100-specific fluorescent probe. Blood was collected 8 h post-dose and plasma was analyzed by the hybridization–ligation assay. **c** Time course of HeLa cell uptake of Cy5.5-labeled PNT100 or encapsulated Cy5.5-labeled PNT100 (representative of PNT2258) monitored by light phase and confocal microscopy

PNT100 and PNT2258 were shown to be stable in whole blood for at least 24 h at 37 °C. Detergent addition and heating samples at 90 °C were required prior to being able to detect PNT100 in PNT2258 by the hybridization–ligation method. Further, no degradation of spiked PNT100 or PNT2258 was observed with long-term storage at −80 °C (>200 days). In effect, no unencapsulated PNT100 is detected in plasma obtained post-intravenous administration. Pretreatment of plasma or tissue samples post-IV administration to release PNT100 from PNT2258 suggests that the nanoparticle remains intact. Similar findings were noted with PNT100, PNT2258, or PNT2258cy (encapsulated cynomolgus monkey-specific sequence of PNT100) visualized by SYBR Green post-24 h exposure in whole blood (Nanotechnology Characterization Laboratory (NCL), unpublished results). Dose-proportional plasma exposure in BALB/c mice treated with 3, 10, or 20 mg/kg PNT2258 was seen with area under the curve values of 22,377, 219,986 or 1,588,000 ng h/mL and *C*_max_ values of 16,585, 37,250 or 151,088 ng/mL, respectively. Half-lives ranged from 2.5 to 9 h. Plasma concentrations for each of the three doses are shown in Fig. 3a. The pharmacokinetic profiles of PNT2258 (20 mg/kg) as a single agent (*C*_max_ 148,331 ng/mL) or in combination with rituximab (*C*_max_ 120,256 ng/mL) in SCID mice with Daudi xenografts were similar to the BALB/c profile, demonstrating a consistent pharmacokinetic profile between naïve and xenografted animals.

The tissue distribution of PNT2258 levels measured after homogenization of tissues and quantified using capillary gel electrophoresis to detect the hybridization of PNT100 with its specific fluorescent probe is shown in Fig. 3b. A consistent with pharmacokinetic clearance, at 8 h post-dose the liver (~10 %), spleen (~12 %), and blood represented approximately 40–50 % of the injected dose with the remainder across a broad tissue distribution including uptake in tumors of WSU-DLCL2 tumor-bearing mice. The uptake of PNT2258 into HeLa cells is shown in Fig. 3c. HeLa cells were used to demonstrate PNT2258 uptake because these cells are relatively resistant to PNT2258-induced cytotoxicity, thereby allowing a time course of uptake to be conducted. Based on the qualitative formulation screen of antitumor activity, physicochemical results, ease of preparation, and in vivo tumor cell localization, the lipid composition to encapsulate PNT100 was selected and further optimized.

**Fig. 4:**
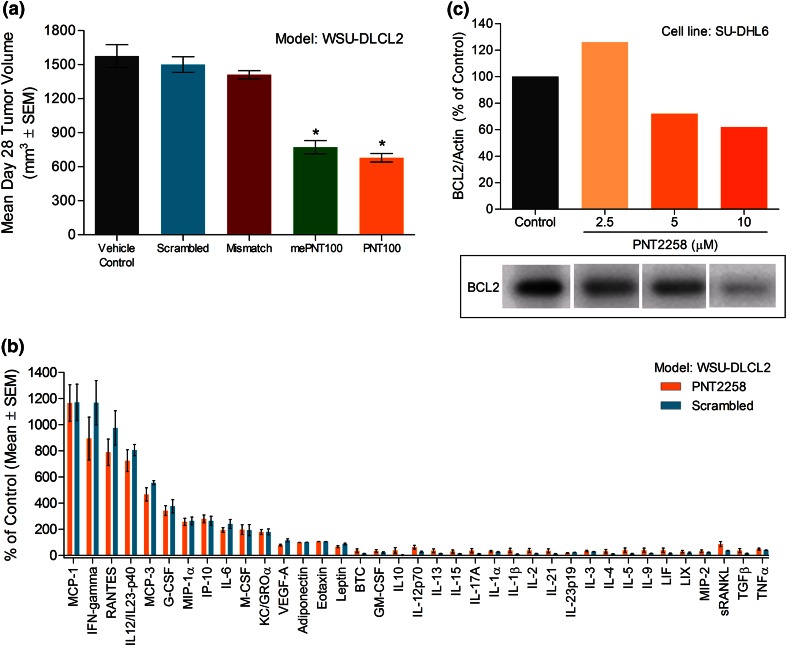
Antitumor activity and plasma immune markers following the administration of PNT100 or control oligonucleotides encapsulated in the lipid composition chosen for PNT2258. **a** Mean tumor volume in SCID mice-bearing WSU-DLCL2 xenografts at 28 days post-tumor implant following IV treatment (10 mg/kg for 5 consecutive days) with PNT100, methylated PNT100, scrambled, mismatched, or vehicle control (*n* = 8 for all groups). *P < 0.05 versus vehicle, scrambled, and mismatched controls. **b** Plasma immune markers in WSU-DLCL2-tumored animals following a single dose of PNT2258 (*n* = 5) or encapsulated scrambled (*n* = 5) control represented as a percent of control (saline-treated *n* = 5). Similar profiles and magnitude of response were seen with PNT2258 and the scrambled control. The only significant difference in marker expression between the two treatment groups was in IFNγ (*P < 0.05). MCP-1, IFNγ, RANTES, IL12/IL23-p40, MCP-3, and G-CSF were all significantly different in both treatment groups compared with the other 31 markers (^♦^ *P* < 0.05). **c** SU-DHL6 lymphoma cells were exposed to PNT2258 at 2.5, 5, and 10 μM. After 72 h of exposure, the contents of triplicate wells were pooled for the BCL-2 protein by immunoblots. The bar graph above the blots represents the densitometric quantification of the BCL-2 protein normalized against actin protein levels

The sequence specificity of PNT100’s antitumor activity in amphoteric liposomes was reconfirmed by demonstrating antitumor activity of PNT2258, but not control oligonucleotides (Fig. 4a). Unmodified oligonucleotides, being natural structures, are quickly degraded and eliminated if not protected (e.g., within a liposomal formulation like PNT2258) and are generally not toxic even at high doses. However, liposomal encapsulation alters the innocuous nature of an unmodified oligonucleotide and published reports suggest encapsulation enhances immunostimulatory properties especially in rodents. While PNT100 contains CpG sequences, it does not contain known motifs that are immunostimulatory. Therefore, to eliminate a concern that immune stimulation was driving PNT100’s BCL-2-targeted antitumor activity, a parallel set of xenografted animals were treated with either PNT2258 or encapsulated scrambled control, and plasma was obtained 24 h post-dose and tested for immune marker modulation (Fig. 4b). PNT2258 and the scrambled control produced similar immune responses in tumor-bearing immune-compromised mice. Markers indicative of innate immune stimulation (IFNγ, IL-6, IL-12p40, IP-10, RANTES) and nanoparticle recognition (MCP-1, MCP-3, G-CSF) were increased following dosing. These findings support a BCL-2-targeted antitumor effect as a result of treatment with PNT2258 and rule out a non-specific immune response driving xenograft antitumor activity. Additional studies exposing PNT100, PNT2258, encapsulated scrambled control, PNT228cy, or empty liposomes to human PBMCs, and probing with a broad immunoplex panel demonstrate modest immune stimulation and are attributed to the recognition of the amphoteric liposome carrier (data not shown and unpublished results, NCL Collaboration). The modulation of BCL-2 following incubation with PNT2258 was confirmed in a SU-DHL-6 lymphoma cell line (Fig. 4c).

### Dose schedule effects of pre-optimized PNT2258 on antitumor activity

**Fig. 5:**
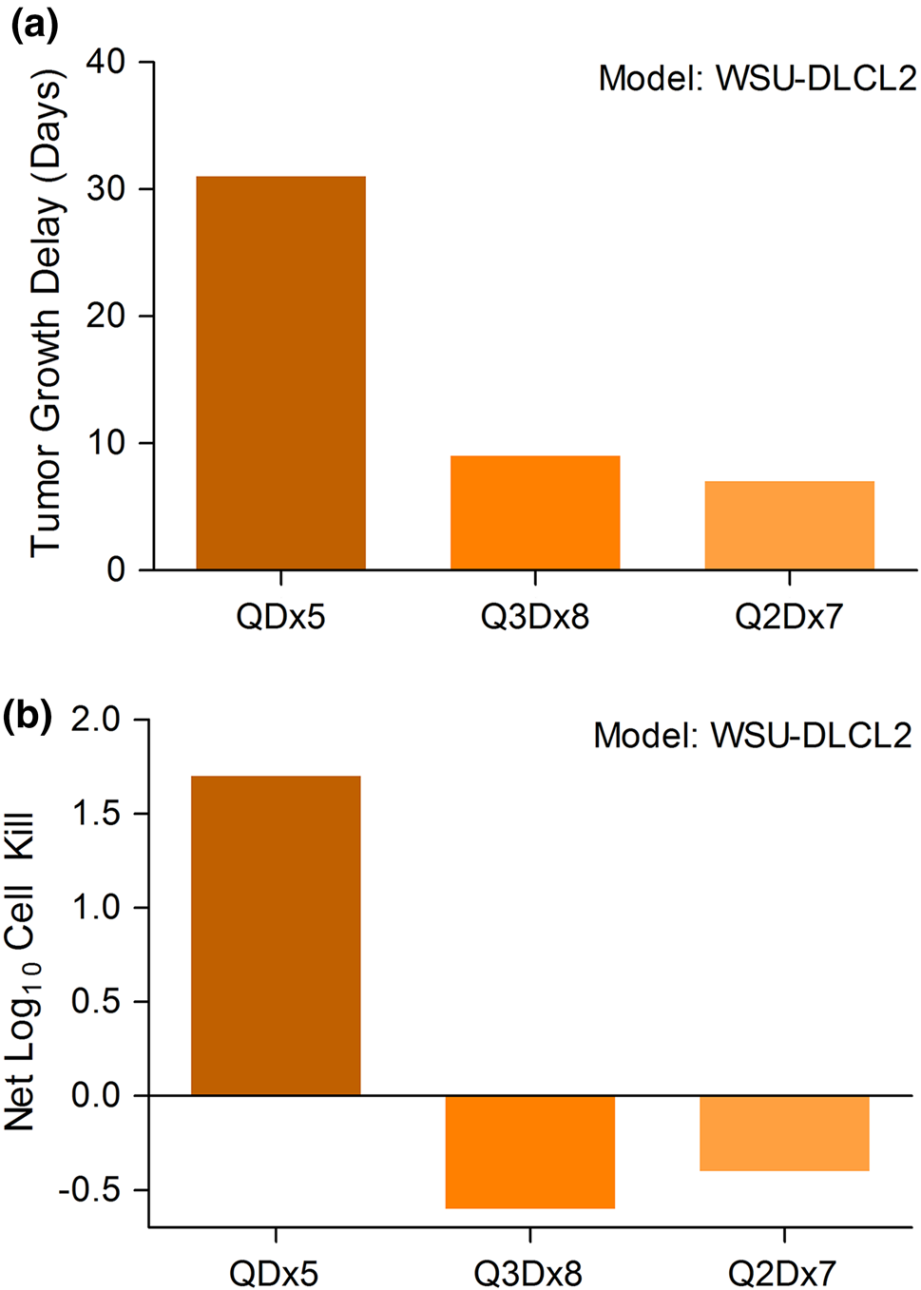
The effect of dose schedule on antitumor activity. **a** Tumor growth delay in C.B-17 SCID mice-bearing WSU-DLCL2 xenografts following administration of 10 mg/kg PNT2258 at the following dose schedules: five IV daily doses (QDx5), 8 IV doses given every third day (Q3Dx8), and 7 IV doses given every other day (Q2Dx7) (*n* = 7 for all groups). **b** Net log_10_ cell kill calculated for each dose schedule. A positive net log_10_ cell kill indicates a decrease, while a negative net log_10_ cell kill indicates an increase, in the tumor cell population at the end of treatment compared with the beginning of treatment

Three treatment schedules, daily for 5 days (QDx5), every other day with seven doses (Q2Dx7), and every third day with eight doses (Q3Dx8), at 10 mg/kg were evaluated against WSU-DLCL2 xenograft tumors (see Fig. 5). Tumor growth delay (Fig. 5a) and net log_10_ tumor cell kill (net kill; Fig. 5b) as a secondary efficacy endpoint were used to compare the activity between treatments. The QDx5 treatment schedule produced a tumor growth delay in excess of 31 days with a corresponding net kill of 1.7 logs, while the tumor growth delay values for the other two treatment schedules were less than 10 days and had negative net kill values, indicating tumor progression. These data suggested the QDx5 treatment schedule was superior and prompted the use of the daily, 5 days treatment schedule in future studies in xenograft studies. The maximum-tolerated dose (MTD) of PNT2258 prototypes was generally 20–30 mg/kg and was xenograft model independent. The MTD is defined at the dose that produces less than 20 % weight loss and is less than or equal to the LD10 in the experimental animals. Weight loss induced by PNT2258 was dose responsive, but never exceeded 20 %. Further, in other testing paradigms, additional treatment cycles were added so that the experimental animals received PNT2258 5 days per week for three consecutive weeks. No dose-limiting toxicities, assessed by body weight changes, were noted between groups.

### PNT2258 activity across tumor xenograft models

**Fig. 6:**
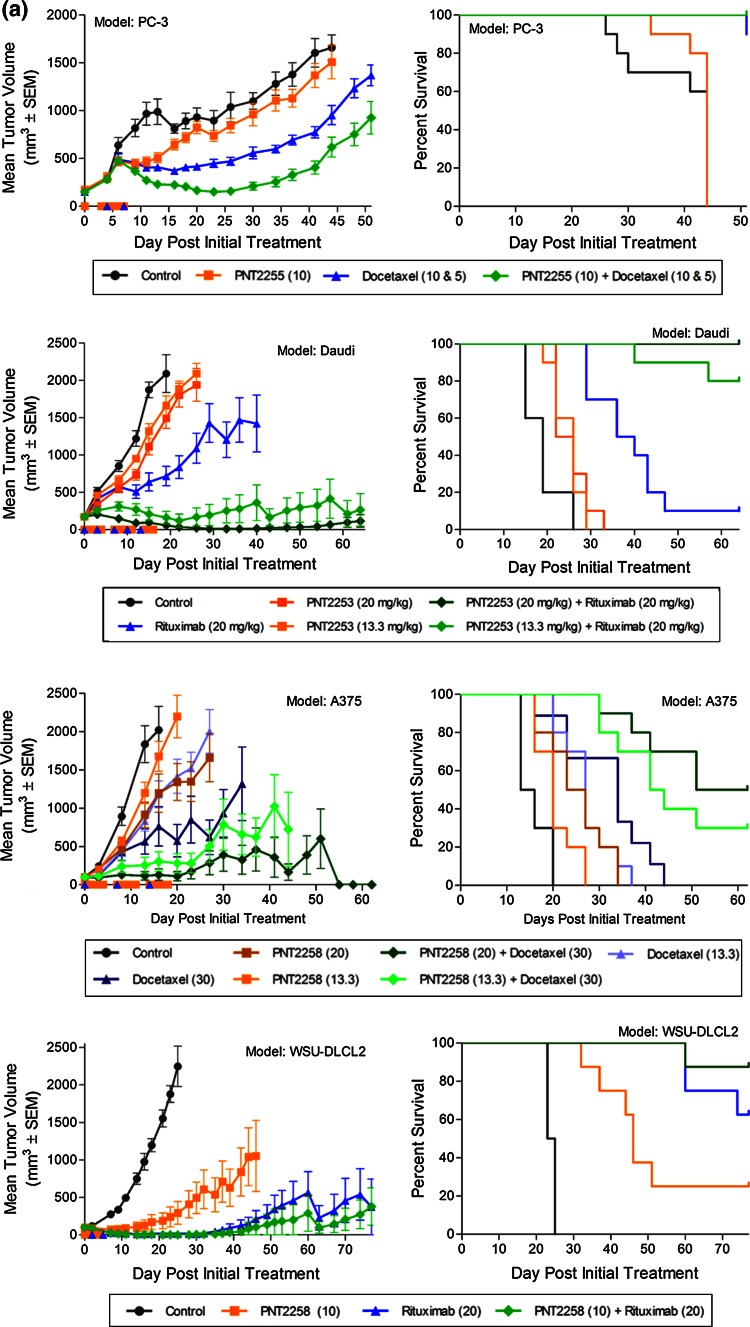

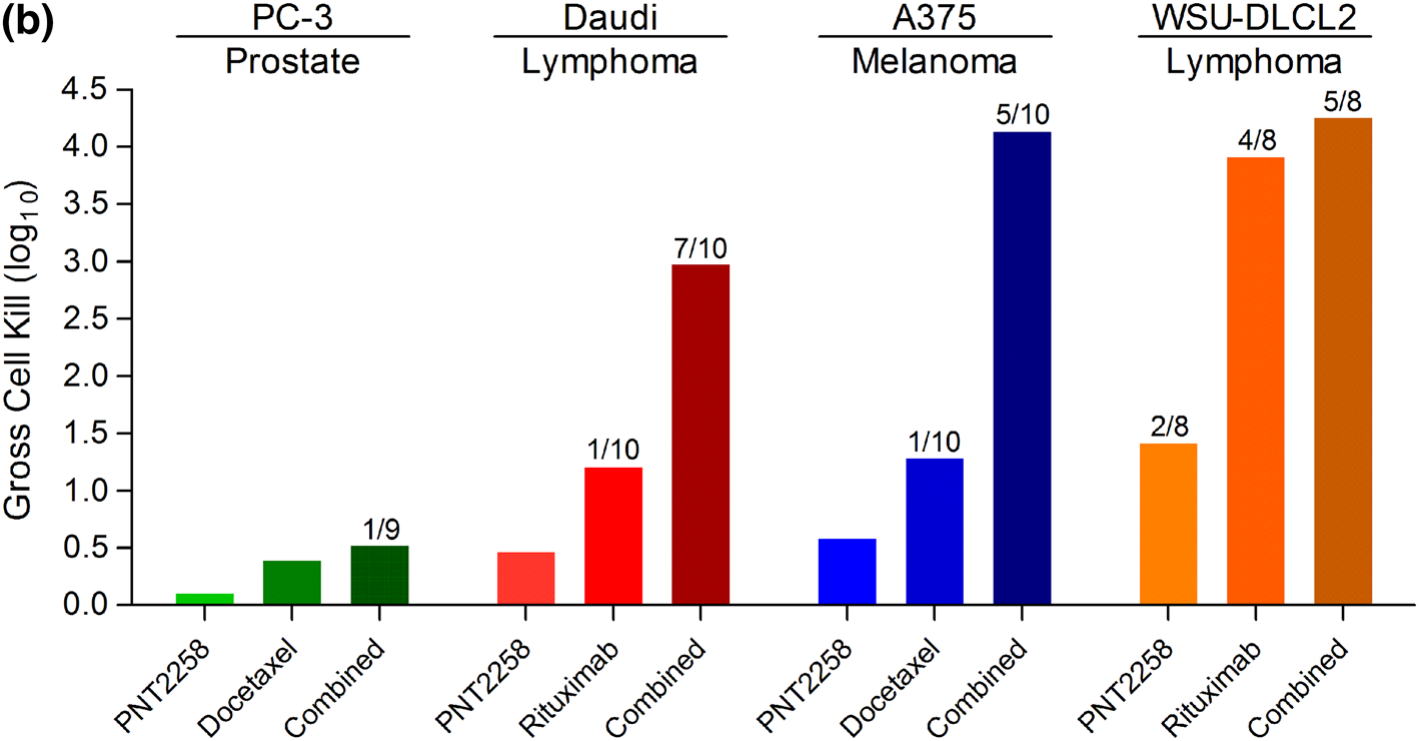
Antitumor effect and percent survival of animals treated with PNT2258 or combination agent as single agents or in combination across four xenograft models. **a** PNT2258 or combination agent was tested against PC-3 (prostate, *n* = 10/group), Daudi-Burkitt’s (lymphoma, *n* = 10/group), A375 (melanoma, *n* = 10/group), and WSU-DLCL2 (lymphoma, *n* = 7/group) alone or in combination. *Symbols* on the *x*-axis represent days of dosing for each treatment; the same schedules were used in combination. The numbers in parentheses after each compound represent the dose (mg/kg) administered. All treatments were administered IV. In the PC-3 study, docetaxel was administered at 10 mg/kg for the initial dose and then at 5 mg/kg for the subsequent dose. **b** Gross cell kill (log_10_) across all four xenograft models. Numbers above the*bars* represent the tumor-free survivors per total animals in the treatment group. The lack of values above a bar indicates that there were no tumor-free survivors for that treatment group

The activity of PNT2258 was tested as a single agent or in combination with docetaxel or rituximab across four xenograft models, WSU-DLCL2 lymphoma, Daudi-Burkitt’s lymphoma, PC-3 prostate, and A375 melanoma and are summarized in Fig. 6a and Supplemental Table 1. WSU-DLCL2 xenografts are representative of a lymphoma cell line harboring the t(14;18) rearrangement with constitutive NF-κB activation driving not only proliferation, but also increased BCL-2 transcription. Daudi-Burkitt’s xenografts harbor a (t8;14) rearrangement and is primarily a CMYC-driven lymphoma subtype. PC-3 xenografts represent a radioresistant model, attributed to increased BCL-2 transcription [35]. A375 is a human-derived melanoma cell line harboring a BRAF mutation due to the substitution of valine for glutamic acid at codon 600, termed V600E resulting in constitutive activation, aggressive proliferation, and high BCL-2 expression. The tumor growth curves for control (saline), PNT2258, docetaxel, or rituximab as single agents or combination treatments are presented in the left panels, with overall survival shown in the corresponding right panels. Generally, weight loss induced by PNT2258 was dose dependent, but average treated body weights did not decrease below 20 % of baseline at any dose as a single agent or in combination with cytotoxic agents.

To compare the activity across models, the data are also represented as gross cell kill (Fig. 6b). Models are represented from left to right based on the single-agent activity of PNT2258 (leftmost bar of each set), and interestingly, an alignment with literature reported BCL-2 protein expression [1, 37, 38]. In some models, there was significant positive interaction between PNT2258 and standard agents (Daudi-Burkitt’s and A375). In the PC-3 and DLCL2 models, the interaction was less pronounced. The combination agents were administered at maximum-tolerated doses reported in the literature for each model and based on the testing facilities’ historically experiences. This dosing paradigm, instead of using suboptimal combination doses, may account for the lack of synergistic or additive effect in the DLCL2 model because the combination agent was so effective. This is supported in other studies (not shown), where synergistic activity of PNT2258 with docetaxel and rituximab was observed when these agents were used at suboptimal doses. The robust antitumor activity provided the rationale to move PNT2258 further into toxicology evaluation and subsequent clinical development.

## Discussion

Oligonucleotide candidates modulating gene expression may be targeted at the level of either RNA or genomic DNA. Approaches to modulate genomic DNA include triplex, quadruplex oligonucleotides, methylated forms of DNA or RNA, or mismatched or single-base modifications of the DNA or RNA oligonucleotides. These methodologies hybridize to transcription start sites to interfere with transcription, cause DNA cleavage, homologous recombination or act to stimulate inherent DNA repair mechanisms to correct the mismatches triggered by the hybrid double-stranded DNA/DNA or chimeric RNA/DNA interactions [38, 39]. To our knowledge, none of these DNA-targeted approaches have progressed into the clinic. In contrast, single- or double-stranded RNA-targeted oligonucleotide approaches have progressed to the point of providing clinical success reviewed in [40] and [41]. These include antisense or siRNA that cause mRNA cleavage and disruption of the RNA translational machinery, RNA modulation agents that correct gene defects by exon skipping, or microRNA miRs and antimiRs agents that regulate the expression of multiple pathways by replacing absent sequences or antagonizing sequences, respectively. A common feature among these approaches is the use of chemical modifications (e.g., phosphothioate, 2’-modified nucleic acids including MOE modifications and cET, conformationally restricted nucleic acids bases including LNA and inverted bases) to enhance activity, pharmacokinetics, and pharmacodynamics [reviewed in [42] ]. In addition, in some cases, the active RNA sequences are not 100 % complementary to their target mRNA.

The 24-base BCL-2-targeted oligonucleotide, PNT100 represents a new class of DNA therapeutics being developed that is distinct from other nucleic acid approaches. PNT100 is an unmodified phosphodiester DNA sequence that is 100 % complementary to its homologous sequence of genomic DNA. Our data demonstrate equivalent antitumor activity of mePNT100 and unmethylated PNT100, but no antitumor effects with control scrambled, randomer, or reverse complement sequences. Moreover, in cellular studies, PNT100’s antiproliferative activity was not affected by a methyltransferase inhibitor, suggesting that methylation is not required for activity. Virtually identical immune response to PNT2258 and its scrambled control in xenografted animals suggests that PNT100’s sequence specificity and not immunogenicity drives BCL-2 modulation and antitumor activity. It is recognized that there are limitations to truly defining control sequences for PNT100. As such, there is the possibility that interactions between the liposomes and specific oligonucleotide sequences may be responsible for the differential effects seen. However, if these effects exist, they should be dose dependent and should have been observed at doses of 10 mg/kg encapsulated oligonucleotide, a dose level that shows antitumor activity for PNT2258. Therefore, by demonstrating, the specificity of PNT100’s pharmacodynamic activity using two independent liposomal systems compared to empty liposomes, and no effect with encapsulated control sequences suggests this is unlikely.

We propose that using unmodified sequences complementary only to a target gene region may offer advantages. The sequence of PNT100 does not possess toll-like receptor (TLR) immunostimulatory CpG motifs, a property that precludes the need to use chemical modifications to mask immune recognition. Further, the observation that PNT100 cannot be measured unless the liposomes are disrupted suggests fully encapsulated PNT100 may also contribute to immune avoidance. Indeed, no significant changes in immune-stimulatory cytokines or clinical signs of anaphylaxis observed following dosing of PNT2258 in patients with advanced solid tumors lends support to this approach [27]. Clinically, dose-dependent BCL-2-targeted effects were observed including reductions in lymphocyte counts [27]. PNT2258 was well tolerated in the clinic with doses up to 150 mg/m^2^ (equivalent to ~4 mg/kg).

The lipid composition used for PNT2258 with its amphoteric pH-tunable nature has several distinct features. First, the overall particle charge is anionic at blood pH which prevents aggregation with blood components and eliminates the need for PEGylation. Second, the composition is sterol rich with CHEMS and MOCHOL, providing the cholesterol backbone to anchor the pH responsive headgroup compositions. Third, ether linkages often used to anchor polyethylene glycol (PEG) or pH responsive headgroups are not used. Finally, the amphoteric nature enables a transition of charge from cationic to net neutral to anionic or vice versa depending on the microenvironment [24, 26]. For example, during transient acidic ethanolic conditions used during formulation, there is an overall positive charge to the mixture enabling the efficient encapsulation of the negatively charged PNT100. A shift to pH 7.5 renders an overall anionic charge to the particle, repelling unencapsulated oligonucleotide, which is removed during ultrafiltration. The PNT2258 drug product typically has a zeta potential of −40 mV. The anionic surface characteristics and unique lipid composition enables stable encapsulation of the oligonucleotide, without surface PNT100, thereby obviating the need for PEG spacers to prevent aggregation or immune recognition. Additionally, the anionic nature likely alters opsonin adhesion or activation (e.g., complement factors) compared with cationic carriers [43]. It may also attract exchangeable apolipoproteins such as apoE to facilitate cellular uptake through lipoprotein and/or other receptor-mediated uptake pathways [44 and references therein]. The novel lipid MOCHOL has been shown to have a pKa of approximately 6.5 [26], which is within the range identified for facilitating endosomal escape [44]. We believe the unique features of the lipids and the ratios of the liposomal system permits PNT2258, under the appropriate pHs and ionic environments to facilitate endosomal escape, a key feature enabling PNT100 access to nuclei.

PNT2258 exhibits good systemic exposure following intravenous administration and demonstrates antitumor activity against xenografted tumors. Our working hypothesis centered on the assumption that BCL-2 expression is generally low in non-cancerous cells and entry of PNT100 into non-cancerous tissue would not interfere with normal homeostasis. Similarly, in tumor types driven by BCL-2 transcription, PNT2258 should demonstrate good single-agent activity (compare WSU-DLCL2 with Daudi-Burkitt’s), but should potentiate the combination drug in tumor types with high BCL-2 expression if BCL-2 resistance is implicated in disease resistance (e.g., Daudi, PC-3, and A375). The key challenge was to achieve sufficient systemic exposure while avoiding dose-limiting lipid toxicity. Therefore, an overriding goal was to ensure that sufficient lipid doses (>100 mg/kg lipid, equivalent to 4 mg/kg PNT2258) could be safely administered to animals to overcome the reticuloendothelial system (RES) clearance mechanisms and enable sufficient exposure to be efficacious without causing observable toxicities. Lipid particle numbers are proportional to the nanoparticle diameters [45] and both these parameters greatly influence circulation lifetimes [46], hepatic uptake [47], and importantly, tolerability and access to extrahepatic tissues in the absence of targeting ligands [48, 49]. Moreover, the pharmacokinetics and tissue access of liposomes are also influenced by surface charge and recognition which are a function of particle size and composition [46]. Liver fenestrae and sinusoids across species represent physical barriers of ~100 nm such that nanoparticles with an average diameter size range of ~130 nm will be primarily removed by resident macrophages and will not readily access hepatocytes, which represent the population of liver cells to which lipid toxicity is attributed [47]. As a result, the liver is generally resilient and can tolerate lipid doses of 100 mg/kg or greater, depending on the rate of delivery, as evidenced by the safety and tolerability of parenteral nutrition containing daily lipid doses of up to 60 g (administered at 2.5 g/h). These factors were taken into account during the development of PNT2258 to ensure that a broad therapeutic window could be identified. This is evidenced by the relatively flat dose response of PNT2258 above 10 mg/kg (EC50 estimated to be between 3 and 10 mg/kg across the models) and tolerability with daily doses of 30 mg/kg (or 750 mg/kg lipid). We speculate that the unique mixture of lipids (that lack ether linkages or PEG) and particle size of the amphoteric liposomes contributes to the therapeutic activity of PNT2258 in several ways. These include imparting stability [24, 26], surface characteristics which prolong circulation times in blood, and permit metabolic breakdown to enable repeated dosing. Toxicology findings in preclinical studies with PNT2258 showed dose-dependent toxicities that were reversible, and attributable to high doses of lipid (>300 mg/kg) rather than PNT100 [27].

PNT2258 shows broad activity against a variety of tumor types with robust single-agent activity in DLBCL where BCL-2 transcription drives the genesis and survival of the tumors. Antitumor activity and long-term survival in combination with docetaxel or rituximab in chemo-resistant tumors were also demonstrated. BCL-2 overexpression is linked to lower overall survival and adversely influences progression-free survival in subtypes of chemotherapy naïve NHL patients [50]. After front-line R-CHOP therapy, 30–50 % of these patients fail to respond, with BCL-2 expression remaining high in patients with germinal center DLBCL, suggesting the need for BCL-2-targeted interventions. High-risk patients with revised International Prognostic Index (R-IPI) scores of 3–5 or those with double-hit (BCL-2/MYC positive) phenotypes demonstrate even worse prognosis with short survival timelines [51]. Moreover, there is a high correlation between BCL-2 expression and the presence of the t(14;18) translocation. Our preclinical data show that PNT2258 demonstrates good single-agent activity and show additive effect with rituximab in DLBCL where the t(14;18) rearrangement exists (WSU-DLCL2) and synergistic activity against Daudi-Burkitt’s, suggesting that targeting BCL-2 can potentiate other therapies. Early clinical data indicate antitumor effect in patients whose tumors may be BCL-2 dependent [27]. Similarly, combination with docetaxel results in an additive effect in prostate and melanoma models. These results support the rationale for combining PNT2258 with approved agents such as docetaxel and rituximab to potentiate their cytotoxic activity in tumor types where BCL-2 plays a role in resistance.

## Electronic supplementary material

Below is the link to the electronic supplementary material.

Supplementary material 1 (DOCX 14 kb)

Supplementary material 2 (TIFF 1533 kb)

Supplementary material 3 (TIFF 450 kb)

Supplementary material 4 (TIFF 1338 kb)

Supplementary material 5 (DOCX 17 kb)
