# Supplementary material for: Development and antitumor activity of a BCL-2 targeted single-stranded DNA oligonucleotide
Source: Cancer Chemother Pharmacol. 2014 May 16;74(1):151–66. doi: 10.1007/s00280-014-2476-y (PMC4077254; doi:10.1007/s00280-014-2476-y)
Supplement: Supplementary file 1 — Supplementary material 1 (DOCX 14 kb) [file 280_2014_2476_MOESM1_ESM.docx]

**Supplemental Figure S1**. Evaluation of BCL2-targeted oligonucleotides cell lines. (A) BCL2-targeted oligonucleotides BL1-BL7 were evaluated in breast cell lines, MDA-MB-231, BT-474, and a melanoma cell line, M14. BL1 and BL7 were also tested in T47D breast cells. Exposure in triplicate wells, were conducted at 10μM for 72 hours followed by analyses of cell viability by MTT assay. BL2 is PNT100. (B) Time course for BCL2-targeted oligonucleotides in WSU-FSCCL cells. BL2 (represents methylated PNT100, mePNT100) and BL7 were exposed in triplicate, at 10μM, and cell viability monitored over the course of 72 hours. (C) Counterscreen of BL2 and BL7 against a nontumorigenic mouse cell line. NMuMG were treated in triplicate at 10μM for up to 96 hours. At 24 and 96 hours post-exposure, cells were counted to measure cell viability and used to assess non-specific activity.
