# Supplementary figures and images for: Development and antitumor activity of a BCL-2 targeted single-stranded DNA oligonucleotide
Source: Cancer Chemother Pharmacol. 2014 May 16;74(1):151–66. doi: 10.1007/s00280-014-2476-y (PMC4077254; doi:10.1007/s00280-014-2476-y)

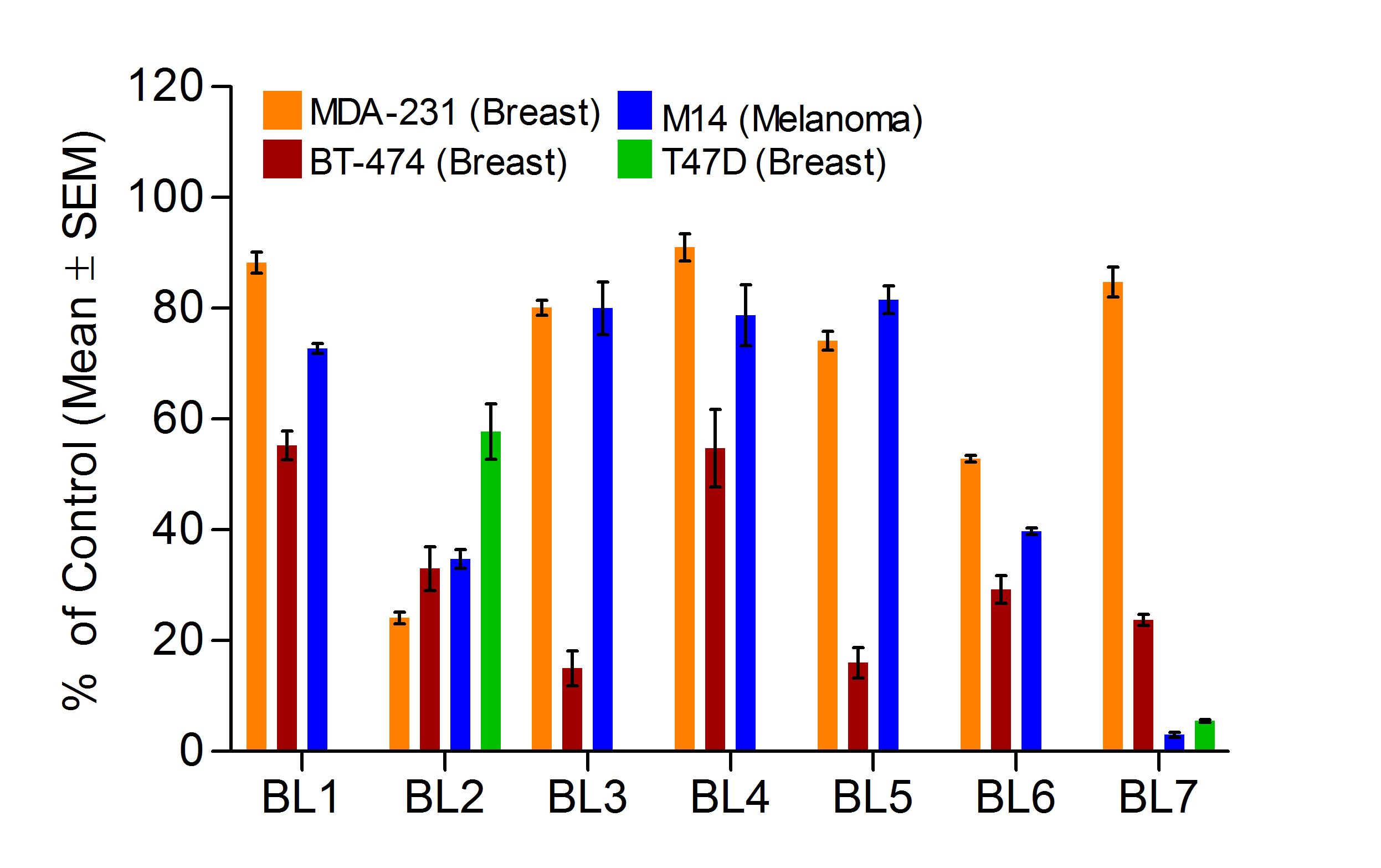

Supplement: Supplementary file 2 — Supplementary material 2 (TIFF 1533 kb) [file 280_2014_2476_MOESM2_ESM.tif]

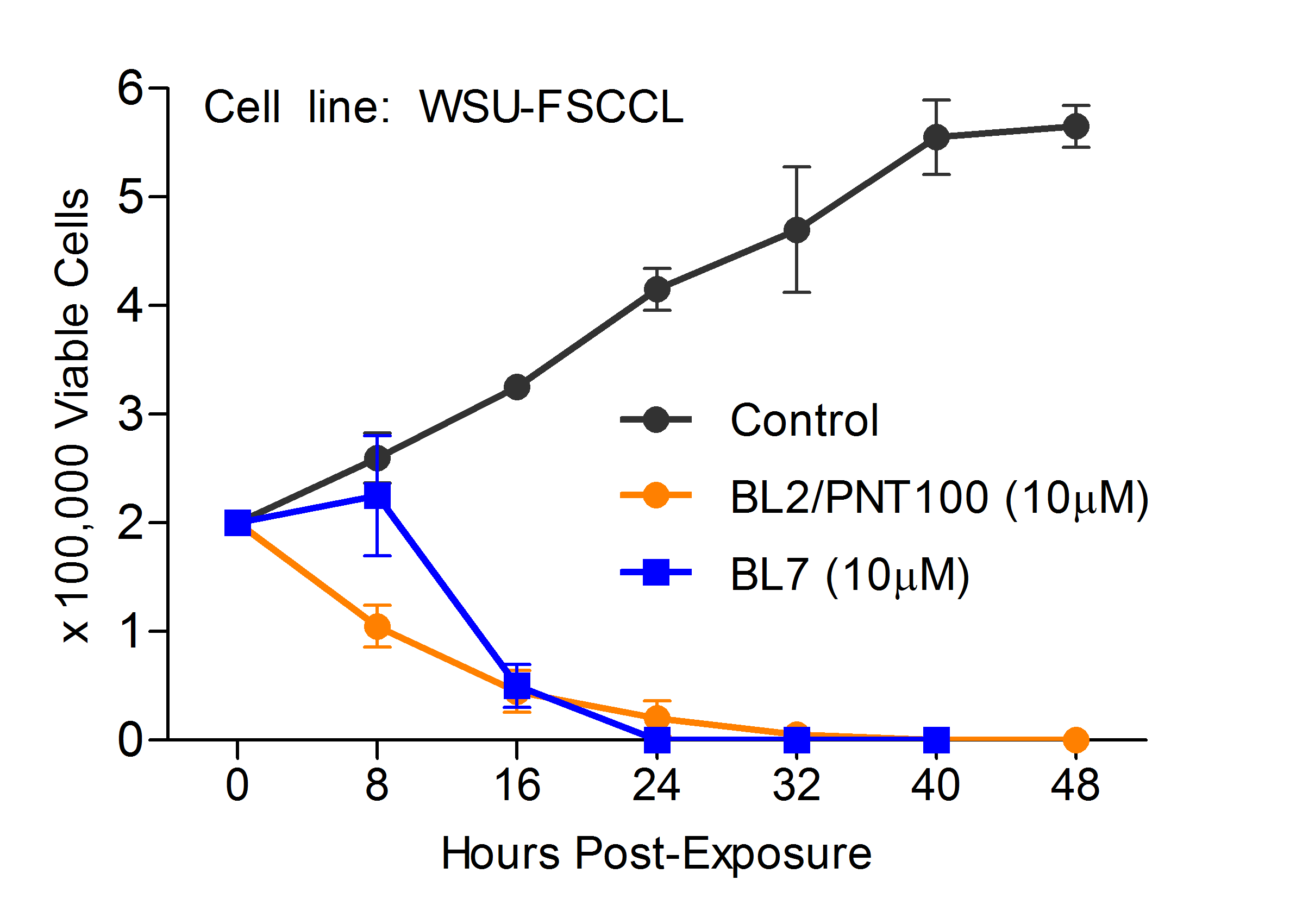

Supplement: Supplementary file 3 — Supplementary material 3 (TIFF 450 kb) [file 280_2014_2476_MOESM3_ESM.tif]

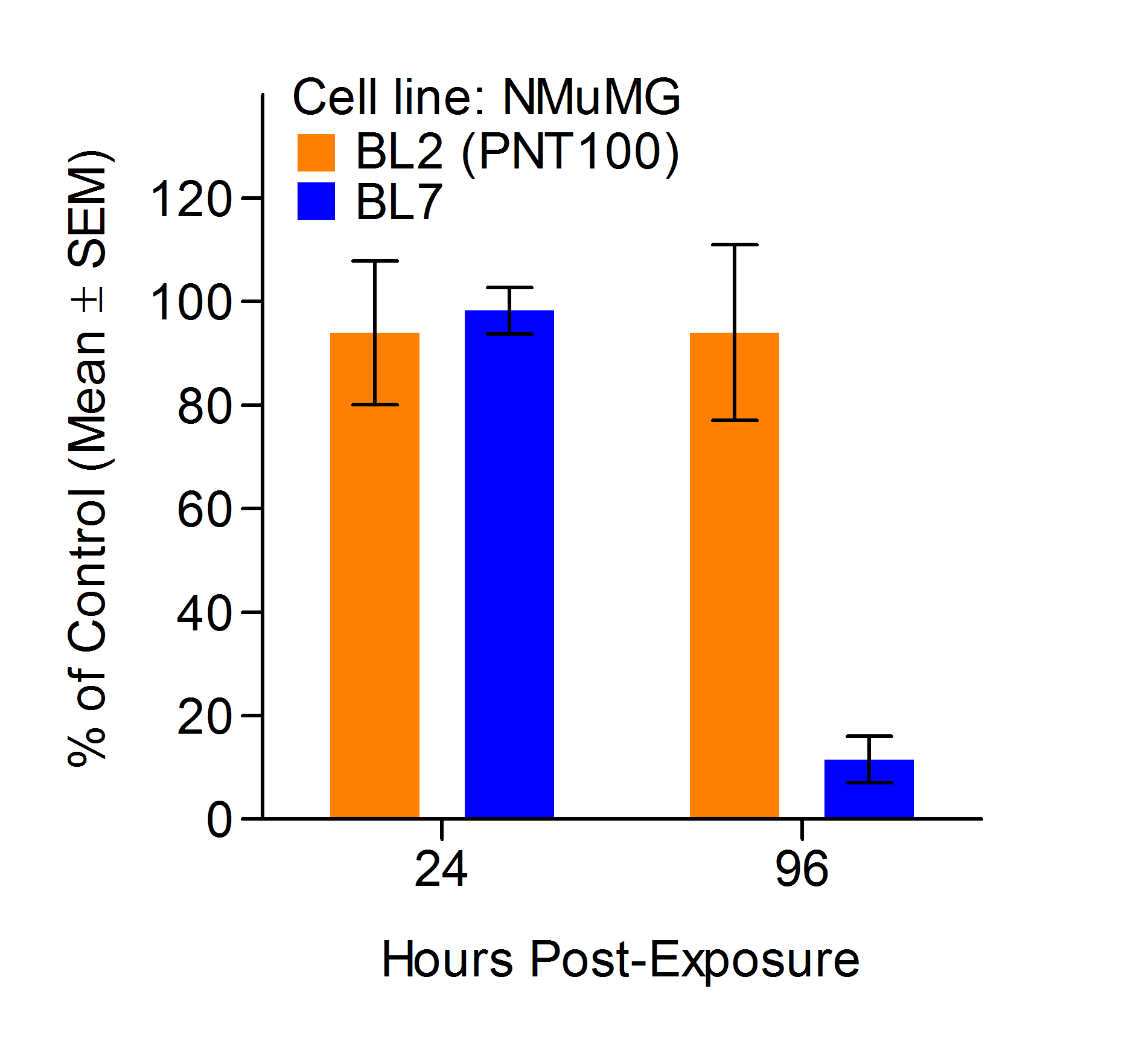

Supplement: Supplementary file 4 — Supplementary material 4 (TIFF 1338 kb) [file 280_2014_2476_MOESM4_ESM.tif]
