# Supplementary material for: Development and antitumor activity of a BCL-2 targeted single-stranded DNA oligonucleotide
Source: Cancer Chemother Pharmacol. 2014 May 16;74(1):151–66. doi: 10.1007/s00280-014-2476-y (PMC4077254; doi:10.1007/s00280-014-2476-y)
Supplement: Supplementary file 5 — Supplementary material 5 (DOCX 17 kb) [file 280_2014_2476_MOESM5_ESM.docx]

Supplemental Table 1: Efficacy summary of PNT2258 in solid and hematological preclinical tumor models

| **Tumor Model** | **Dose  (mg/kg) ^1^** | **PNT2258 Schedule^2^** | **Combo Agent** | **Dose  (mg/kg)^2^** | **Combo Agent**  **Schedule** | **PR  N (%)** | **CR**  **N (%)** | **Tumor Free Survivors**  **N (%)** | **Tumor Growth Delay (days)** | **Net Cell Kill** | **Min % T/C** |
| --- | --- | --- | --- | --- | --- | --- | --- | --- | --- | --- | --- |
| PC-3 | - | - | docetaxel | 10 and 5 | Day 4 & 7 | 0 (0%) | 0 (0%) | 0 (0%) | 21 | 0.3 | 43% |
| PC-3 | 10 | Day 1, Day 3 qd x 5 | - | - | - | 0 (0%) | 0 (0%) | 0 (0%) | 5 | 0 | 57% |
| PC-3 | 10 | Day 1, Day 3 qd x 5 | docetaxel | 10 and 5 | Day 4 & 7 | 0 (0%) | 0 (0%) | 1 (11%) | 29 | 0.4 | 18% |
| Daudi-Burkitt's | - | - | rituximab | 20 | biwk x 2.5 | 0 (0%) | 0 (0%) | 1 (10%) | 20 | 0.2 | 32% |
| Daudi-Burkitt's | 30 | (qd x 5;2d off) x 3 | - | - | - | 0 (0%) | 0 (0%) | 0 (0%) | 6 | 0.2 | 39% |
| Daudi-Burkitt's | 20 | (qd x 5;2d off) x 3 | - | - | - | 0 (0%) | 0 (0%) | 0 (0%) | 8 | -0.6 | 56% |
| Daudi-Burkitt's | 13.3 | (qd x 5;2d off) x 3 | - | - | - | 0 (0%) | 0 (0%) | 0 (0%) | 7 | -0.6 | 71% |
| Daudi-Burkitt's | 8.89 | (qd x 5;2d off) x 3 | - | - | - | 0 (0%) | 0 (0%) | 0 (0%) | 6 | -0.7 | 74% |
| Daudi-Burkitt's | 5.92 | (qd x 5;2d off) x 3 | - | - | - | 0 (0%) | 0 (0%) | 0 (0%) | 7 | -0.6 | 64% |
| Daudi-Burkitt's | 20 | (qd x 5;2d off) x 3 | rituximab | 20 | biwk x 2.5 | 2 (20%) | 0 (0%) | 7 (70%) | >49 | 1.9 | 1% |
| Daudi-Burkitt's | 13.3 | (qd x 5;2d off) x 3 | rituximab | 20 | biwk x 2.5 | 0 (0%) | 1 (10%) | 6 (60%) | >49 | 1.9 | 1% |
| A375 | - | - | docetaxel | 30 | qwk x 3 | 0 (0%) | 0 (0%) | 1 (10%) | 14 | -0.1 | 14% |
| A375 | - | - | docetaxel | 20 | qwk x 3 | 0 (0%) | 0 (0%) | 1 (10%) | 9 | -0.5 | 28% |
| A375 | - | - | docetaxel | 13.3 | qwk x 3 | 0 (0%) | 0 (0%) | 0 (0%) | 7 | -0.7 | 36% |
| A375 | 20 | (qd x 5;2d off) x 3 | - | - | - | 0 (0%) | 0 (0%) | 0 (0%) | 6 | -1.1 | 35% |
| A375 | 13.3 | (qd x 5;2d off) x 3 | - | - | - | 0 (0%) | 0 (0%) | 0 (0%) | 3 | -1.5 | 62% |
| A375 | 8.89 | (qd x 5;2d off) x 3 | - | - | - | 0 (0%) | 0 (0%) | 0 (0%) | 5 | -1.3 | 42% |
| A375 | 20 | (qd x 5;2d off) x 3 | docetaxel | 30 | qwk x 3 | 1 (10%) | 0 (0%) | 5 (50%) | >46 | 2.4 | 1% |
| A375 | 20 | (qd x 5;2d off) x 3 | docetaxel | 20 | qwk x 3 | 0 (0%) | 0 (0%) | 0 (0%) | 12 | -0.6 | 24% |
| A375 | 13.3 | (qd x 5;2d off) x 3 | docetaxel | 30 | qwk x 3 | 0 (0%) | 1 (10%) | 2 (20%) | 25 | 0.5 | 6% |
| A375 | 13.3 | (qd x 5;2d off) x 3 | docetaxel | 20 | qwk x 3 | 1 (10%) | 0 (0%) | 3 (30%) | 11 | -0.7 | 12% |

^1^Dose calculated based on total oligonucleotide content measured by OD260.  ^2^Schedule: qdx5; 2d off=five daily doses then two days off. N=number of animals; PR=partial responder; CR=complete responder.
